# Ter94/VCP Is a Novel Component Involved in BMP Signaling

**DOI:** 10.1371/journal.pone.0114475

**Published:** 2014-12-03

**Authors:** Zhao Zeng, David J. J. de Gorter, Maria Kowalski, Peter ten Dijke, Osamu Shimmi

**Affiliations:** 1 Institute of Biotechnology, University of Helsinki, Helsinki, Finland; 2 Department of Molecular Cell Biology and Cancer Genomics Centre Netherlands, Leiden University Medical Centre, Leiden, The Netherlands; University of Otago, New Zealand

## Abstract

Bone morphogenetic proteins (BMPs), a subgroup of the transforming growth factor (TGF)-β family, transduce their signal through multiple components downstream of their receptors. Even though the components involved in the BMP signaling pathway have been intensely studied, many molecules mediating BMP signaling remain to be addressed. To identify novel components that participate in BMP signaling, RNA interference (RNAi)-based screening was established by detecting phosphorylated Mad (pMad) in *Drosophila* S2 cells. Ter94, a member of the family of AAA ATPases, was identified as a novel mediator of BMP signaling, which is required for the phosphorylation of Mad in *Drosophila* S2 cells. Moreover, the mammalian orthlog of Ter94 valosin-containing protein (VCP) plays a critical role in the BMP-Smad1/5/8 signaling pathway in mammalian cells. Genetic evidence suggests that Ter94 is involved in the dorsal-ventral patterning of the *Drosophila* early embryo through regulating decapentaplegic (Dpp)/BMP signals. Taken together, our data suggest that Ter94/VCP appears to be an evolutionarily conserved component that regulates BMP-Smad1/5/8 signaling.

## Introduction

Understanding tissue generation during metazoan development requires knowledge of how cell and tissue identity is established. Central to this issue is the characterization of signaling molecules, cell-cell interactions, receptors, and second messenger systems that contribute to the processes of cell differentiation and specification. The transforming growth factor (TGF)-β family represents the largest collection of growth factors identified to date and consists of more than 30 secreted polypeptides. Within this family, the bone morphogenetic proteins (BMPs) constitute the largest subgroup. BMPs regulate biological processes as diverse as cell proliferation, differentiation, cell-fate determination, apoptosis, and morphogenesis [1], [2].

TGF-β type ligands activate downstream signaling by binding to their specific membrane receptors. Upon ligand binding, a tetrameric complex of type I and type II serine/threonine kinase receptors forms. The type II receptors then phosphorylate the type I receptors, which subsequently phosphorylate the receptor-regulated Smads (R-Smads): Smad1/5/8 for BMP-type signaling and Smad2/3 for TGF-β/Activin-type signaling. Thereafter, R-Smads form a complex with common Smads (Co-Smads) and translocate into the nucleus, where transcriptional activation or repression of target genes occurs [2]. Ablation of the TGF-β signaling pathway underlies a variety of developmental defects or diseases [3], [4], thus it is crucial to address the mechanism of how the signaling activity is regulated.

In *Drosophila*, seven TGF-β family ligands have been identified, three of which, decapentaplegic (Dpp), screw (Scw), and glass bottom boat (Gbb), are within the BMP subgroup. Two type I receptors, thickveins (Tkv) and saxophone (Sax), and two type II receptors, put (Punt) and wishful thinking (Wit), have been identified to function as BMP receptors in *Drosophila*. Moreover, one R-Smad (mother against dpp; Mad) and one Co-Smad (Medea) were identified as downstream of BMP signaling in *Drosophila*. In *Drosophila,* BMP signals have been implicated in regulating a diverse array of developmental events ranging from dorsal-ventral patterning of the early embryo, oogenesis, mid gut development, the patterning and growth of imaginal tissue, wing vein formation, and synapse function at the neuro-muscular junction [5]–[10]. Studies of TGF-β signaling in *Drosophila* will impact our understanding of TGF-β signaling in mammals, since the fundamental signaling mechanism is highly conserved in both mammals and *Drosophila* [2]. Additionally, the relatively simple signaling pathways in *Drosophila* and the easiness of *Drosophila* genetic analysis make *Drosophila* an ideal model for identifying key determinants in TGF-β signaling.

Many fundamental problems in signal transduction have been studied in tissue culture systems. The recent development of functional analysis of genes by RNA interference (RNAi) in cell culture energized the field of functional genomics by enabling genome-scale loss-of-function screens in cultured cells. Driven by genome sequence data, RNAi can serve in high-throughput screens [11]. Although the basic mechanism for TGF-β type signal transduction has been well established, points of modulation are still poorly understood, indicating that many additional components remain to be discovered.

In this study, in order to identify novel genes that regulate TGF-β type signaling in *Drosophila*, we established a screening system of BMP signaling in *Drosophila*. Genome-wide RNAi screening in *Drosophila* cells revealed Ter94 as a novel component of BMP signaling during embryogenesis. Our data also reveal that the mammalian ortholog valosin-containing protein (VCP) is required for BMP signaling in mammalian cells. These data suggest that Ter94/VCP is an evolutionarily conserved component of the BMP signaling pathway.

## Materials and Methods

### Constructs

Constitutive active promoter pBRAcPA-*dpp-HA*, pBRAcAP-*scw-HA*, and pBRAcPA-*Flag-Mad* for cell cultures have been described previously [12]. Flag-Mad was constructed into a Gateway-compatible vector containing a heat shock inducible promoter (pHFW, Drosophila Genomics Resource Center) or a constitutive active actin promoter (pAFW). Ter94 and human VCP (hVCP) were constructed into a Gateway vector containing a constitutive active promoter (pAW) for cell culture. The following primers were used in the gateway cloning of Ter94 or hVCP:

Ter94∶5′-CACCATGGCAGATTCCAAGGGTGAAGAT-3′ and 5′-CTAACTGTAAAGATCATCGTCGCC-3′.

hVCP: 5′-CACCATGGCTTCTGGAGCCGATTCAAAAGGTGA-3′ and 5′-TTAGCCATACAGGTCATCATCATTGTCTTCTGTG-3′.

As for preparation of dsRNA, pairs of primers containing a T7 sequence overhang (TAATACGACTCACTATAGGGAGA) were designed to amplify the DNA sequences of target genes by PCR. PCR products were used as a template to synthesize dsRNA molecules with an *in vitro* transcription MEGAscript T7 Kit (AM1334, Applied Biosystems). An RNeasy Mini Kit (74104, Qiagen) was used for the purification of dsRNAs, and NanoDrop (Thermo Scientific) was used for dsRNA quantification. The following primers were used in the production of dsTer94-3′UTR:

TER94-3′UTR-F: 5′-TAATACGACTCACTATAGGGAGAGCCTCATCTTGAATTTGACT-3′.

TER94-3′UTR-R: 5′-TAATACGACTCACTATAGGGAGACTAGTTGACGTTGAACTTTT-3′.

### BMP signaling assay and Western blotting

A cell-based BMP signaling assay and RNAi were conducted as described previously with modifications [13]. *Drosophila* S2 cells transfected by pBRAcPA-*Flag-Mad* were incubated with dsRNA. *LacI* RNAi served as a control. Three days after transfection, the cells were incubated with Dpp ligand for four hours. The cells were then spun down and resuspended into 100 µl 1x SDS-PAGE sample buffer. The BMP signals were then measured wtih Western blotting probing with the following antibodies: primary antibodies, mouse anti-Flag M2 (Sigma), mouse anti-Tubulin (Sigma), and rabbit anti-pMad antibodies; secondary antibodies, anti-mouse-680 (LI-COR) and anti-rabbit-800 (LI-COR) antibodies; all were then analyzed with the Odyssey Infrared Imaging System (LI-COR). Anti-Ter94 antibody was obtained from Dennis McKearin [14].

### Primary RNAi screening

#### dsRNA library

The dsRNAi library, which was prepared with *Drosophila* Genome Collections (DGC) 1, 2 and 3 as templates, was provided by the High Throughput Center, University of Helsinki [15].

#### Transfection

In a primary screen, S2 cells were transfected with 40 ng of two dsRNAs, 20 ng of pHFW-*Flag-Mad*, 20 ng of *dpp-HA*, and 20 ng of *scw-HA* in a 384-well plate (3701, Corning). A high throughput Biomek robot served for transfection. In the first part of the primary screen, transfections were performed in a duplicate.

When the dsRNA pairs affected relative intensities of BMP signaling (below or above 80–120% of the control) in the first part of the RNAi screen, 40 ng of individual dsRNA were collected and used in the second part of primary screen. Transit (MIR 2006, Mirus) was used for transfection in the high throughput screen.

#### Staining

Five days after transfection, the cells were incubated for 45 minutes at 37°C and for another four hours at room temperature. Then all the cells in the 384-well plates were suspended by the Biomek robot and transferred to a 384-well view plate (6005261, Perkin Elmer) precoated with 0.05 mg/ml Concanavalin A (Sigma). The cells were then fixed with 3.7% formaldehyde in HBS (10 mM HEPES, 135 mM NaCl, 0.4 mM MgCl_2_, 1 mM CaCl_2_, pH 7.4) for 10 min at room temperature, permeabilized wtih HBST (HBS, 0.1% Triton X-100) for 10 min, and incubated with HBS-B (HBS, 1% BSA) for 30 min. The cells were then incubated overnight with mouse anti-Flag M2 (1∶500) and rabbit anti-pMad (1∶200) at 4°C as primary antibodies, and with Alexa Fluor 488 goat anti-mouse (1∶500), Alexa Fluor 647 goat anti-rabbit (1∶500) and DAPI (Sigma, 1∶1000) for one hour at room temperature as secondary antibodies.

#### Capturing images and data analysis

An Arrayscan 4.5 high throughput microscope served for capturing images. Three different images in one field were taken to capture DAPI, Flag-Mad and pMad signals. Images of 25 fields were taken for each well. The Arrayscan program served to count the number of nuclei (DAPI), Flag-Mad positive cells, and pMad positive cells, as well as to calculate the relative intensities of the BMP signal in each sample.

### Secondary RNAi screening

#### Preparation of dsRNA

For those genes regarded as hits in the primary screen, the corresponding bacteria strains were collected from the DGC plasmid library and cultured for preparing DNA templates for PCR. PCR was performed with T7 overhang primers, and verified PCR products were stored at 100 ng/µl in a 96-well plate for future usage.

#### Transfection/staining

Three independent transfections in 96-well plates were performed in a secondary screen with modified protocols of the primary screen.

### Tertiary RNAi screening

Those genes regarded as hits in the secondary screen were underwent further analysis. S2 cells were transfected with 200 ng of *pAFW-Mad* and 400 ng of dsRNA in a 96-well plate. Five days after transfection, the cells were incubated with purified Dpp (159-DP/CF, R&D System). The numbers of nuclei, Flag-Mad positive cells, and pMad positive cells were measured with modified protocols of the primary screen in three independent experiments.

### BMP signaling, luciferase assay in mammalian cells

#### Materials

Recombinant BMP6 was kindly provided by Prof. Dr. S. Vukicevic.

#### Cell culture

Mouse pluripotent mesenchymal KS483 cells [16], [17] were cultured in αMEM (GIBCO) and mouse pre-myoblast C2C12 cells [17] in DMEM (GIBCO), supplemented with penicillin/streptomycin (Invitrogen) and 10% Fetal Bovine Serum (FBS) (Integro).

#### ALP and luciferase activity assays

C2C12 cells were transfected with Mouse ON-TARGET plus siRNAs targeting VCP, Smad1 or control siRNAs (Dharmacon) and stimulated with BMP6; alkaline phosphatase (ALP) activity was determined subsequently essentially as described previously [17].

KS483 cells were transfected in 12-well plates with 0.15 µg of firefly luciferase reporter construct, 0.05 µg of a *LacZ* expression plasmid together with 0.30 µg of a VCP or control expression plasmid per well essentially as described previously [17].

### *Drosophila* stocks and in situ hybridization

*dpp^H46^* or *dpp^hr4^* served as a null or hypomorphic allele, respectively. *Ter94^k15002^, Ter94^EY03486^, Ter94^03486^, Ter94^26-8^, Ter94^22-30^* and *tkv^8^* were obtained from Bloomington. *Smurf^15c^* or *Mad^12^* was from Chip Ferguson or Stuart Newfeld, respectively. The *in situ* hybridization of *race* to whole-mount embryos was performed with digoxigenin-labeled RNA probes and visualized with alkaline phosphatase precipitates as described previously [12]. Mutant embryos were identified by a lack of hybridization of *lacZ* transcripts produced from the Cyo, ftz-lacZ balancer chromosome.

### Statistical analysis

All results were expressed as the mean ± s.d. The Student’s *t*-test served for statistical analysis, and P<0.05 was considered statistically significant.

## Results

### Genome-wide RNAi screening is established for BMP signaling in *Drosophila* S2 cells

**Figure 1:**
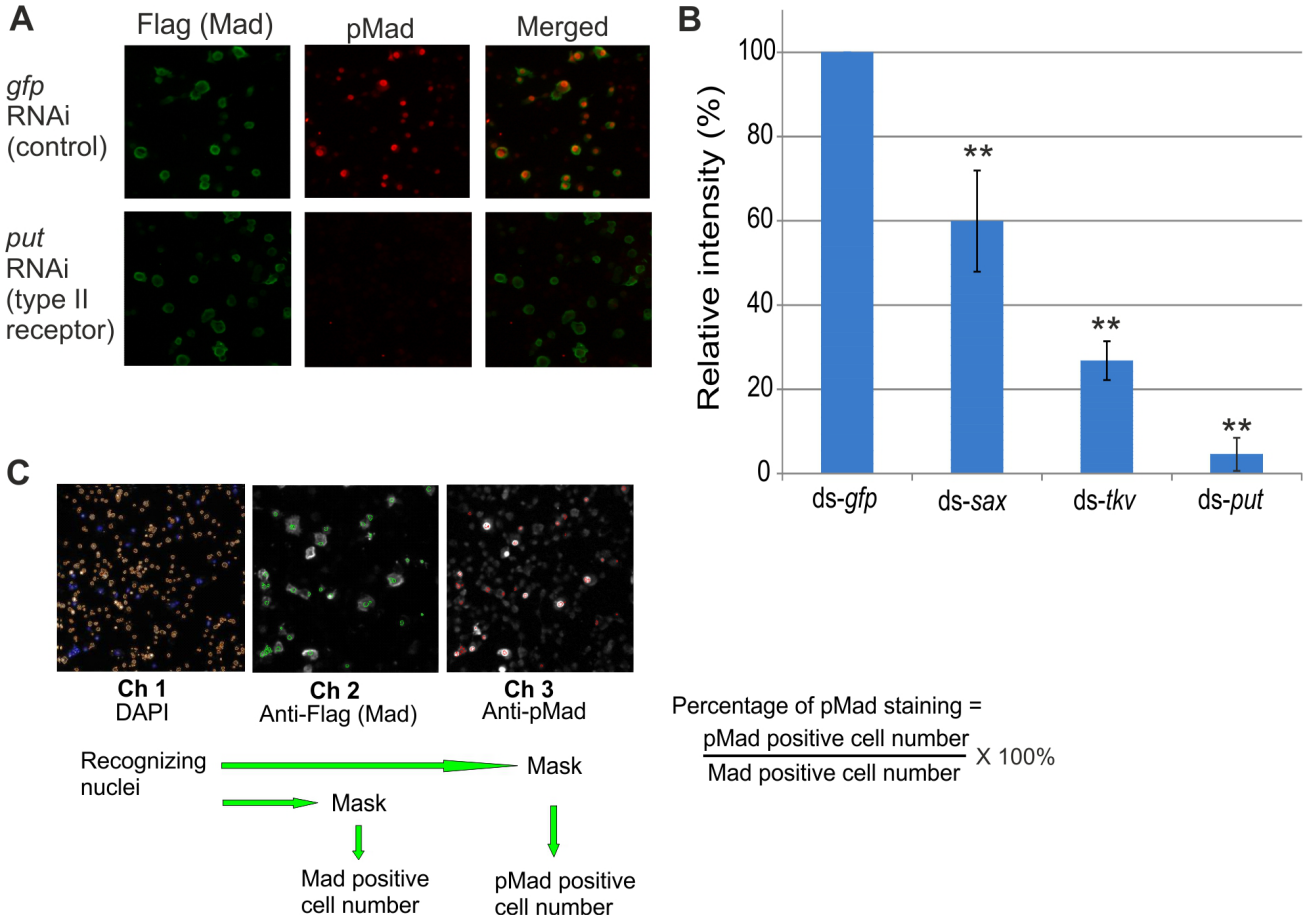
Imaging-based assay of BMP signaling for RNAi screening in *Drosophila* S2 cells. (A) Imaging-based analysis of BMP signals in *Drosophila* S2 cells. Dpp, Scw and Flag-tagged Mad were co-transfected in S2 cells together with dsRNA in 384-well plates. Mad expressions were detected with anti-Flag/Alexa 488 anti-mouse IgG antibodies, and BMP signals were detected with anti-pMad/Alexa 647 anti-rabbit IgG antibodies. dsRNA against *gfp* served as a control to rule out any nonspecific effects caused by RNAi transfection, and the BMP type II receptor *put* served to validate that its knockdown inhibits pMad expression. (B) Imaging analysis of the BMP signals in S2 cells reflects the relative intensities that were regulated by various RNAi approaches (ds-*gfp*, ds-*sax*, ds-*tkv*, and ds-*put*). The relative intensities of BMP signals by RNAi from four independent experiments were measured with imaging-based analysis. The intensity of ds-*gfp* was set at 100%. ** indicates P<0.01 (ds-*sax*, ds-*tkv*, or ds-*put* was compared to ds-*gfp*). (C) The protocol for genome-wide RNAi screening of BMP signaling. The Arrayscan program enabled to calculate the number of DAPI (channel 1) as a total cell number; the number of Flag-Mad-positive cells (channel 2) within the DAPI-positive cells was counted as the Mad-positive cells, and then the number of pMad-positive cells (channel 3) within the Flag-positive cells was counted as the pMad-positive cells. The relative intensity of each sample was calculated as a percentage of pMad staining against Mad (Flag)-positive cells.

Previous studies have shown *Drosophila* S2 cells to be suitable for detecting the BMP signal in a ligand dose-dependent manner and for performing a functional analysis of signaling components by RNAi [13], [18]. In such cases, the readout of phosphorylated Mad (pMad) by Western blotting with anti-pMad antibody enables the system to measure BMP signaling activity. To investigate the BMP signaling pathway in S2 cells through systematic RNAi, an imaging-based method was developed. *Drosophila* BMP-type ligands Dpp and Scw, and Flag-tagged Mad were cotransfected together with dsRNA in S2 cells and incubated for five days, after which images of both Flag-tagged Mad (Mad expression) and pMad staining were captured. To assess the effects of RNAi in the imaging-based assay, dsRNA against *gfp* served as a control to rule out unspecific effects caused by RNAi transfection, and RNAi against the BMP type II receptor *put* served to validate that depletion of upstream signaling components inhibits pMad expression. When *gfp* RNAi was transfected, most of the cells expressing Flag-Mad showed pMad accumulation in the nucleus. In contrast, the pMad was significantly reduced when *put* was knocked down by RNAi (Fig. 1A). To quantify BMP signaling activity, the total number of Flag-Mad expressing cells and the number of pMad-positive cells within the Flag-Mad positive cells were counted, and the ratio of pMad- versus Flag-positive cells was calculated as a relative intensity of BMP signaling. BMP type I receptors, *tkv* or *sax*, or *put* were knocked down in S2 cells, and the relative intensities of their BMP signaling activities were examined. Reductions in BMP signals upon the knockdown of each BMP receptor were consistent with previous results that had been analyzed with Western blotting; *put* knockdown proved to be more efficient than *sax* or *tkv* depletion (Fig. 1B) [13]. This result indicates that the imaging-based analysis of *Drosophila* S2 cells appears to be a suitable system for RNAi screening. To optimize the conditions for large-scale screening, images of cells stained with Flag and pMad antibodies in 384 multiwell plates were obtained with the high throughput fluorescent microscope Arrayscan, and the protocol was established for detecting BMP signals in S2 cells (Fig. 1C). The pMad score was obtained for each well that was transfected with dsRNA. In a pilot screening, the pMad scores were measured when ds-*gfp*, ds-*put*, ds-*sax*, ds-*tkv* RNAs or randomly selected dsRNAs were incubated with S2 cells on 384 plates. RNAi analyses of *gfp*, *tkv*, *sax*, and *put* showed consistent results in small-scale studies, suggesting that imaging-based analysis is feasible for large-scale screening.

We studied about 12 000 dsRNAs consisting of around 80% of annotated genes in *Drosophila* as a primary screen performed in two different parts. In the first part, two randomly selected dsRNAs were combined and examined in each well of the 384 multiwell plates and their effects on BMP signaling intensities were measured. The high throughput microscope served to measure the pMad score by calculating the percentage of pMad-positive cell number versus Mad-positive cell number (Fig. 1C). ds-*gfp* transfected cells served as a control for dsRNA transfections, and their pMad score was set to 100% as the relative intensity of BMP signals. The signal thresholds were set at 80–120% of the control, and genes below or above these ranges were selected as putative candidates. Then, in the second part of the primary screening, individual RNAi selected as putative candidates were examined with the same protocol. This resulted in 447 clones as hits, among which were the components of the canonical BMP signaling pathway, such as Mad, Tkv, and Put (Table S1).

Further analysis of the candidate genes entailed subsequent secondary screening. The assay was repeated three times in 96-well plate format to identify candidate genes that are potentially involved in BMP signaling in S2 cells. The *gfp* RNAi again served as a control in each plate. Secondary screening confirmed that 148 hits induce significant changes in the relative intensities of BMP signaling (below or above 80–120% of the control) (Table S2).

Tertiary screening served to further confirm whether the candidate genes are involved in BMP signaling in S2 cells. To characterize the specificities of candidate genes in BMP signaling, both the pMad score and cell numbers were counted. When a hit resulted in a lower or higher pMad score, a normal Mad score, and a normal cell number, it remained on the hit list. When a hit resulted in a lower or higher pMad score and a normal cell number, but a lower Mad score, it also remained on the hit list. When a hit resulted in a lower pMad score, but a lower Mad score and cell number, it was considered as a common factor involved in cell viability and excluded from the hit list. A summary of the candidate genes listed after tertiary screening appears in Table 1. We identified RNAi of 16 genes (18 clones), resulting in loss-of-function phenotypes in BMP signaling, and RNAi of one gene MAN1, resulting in a gain-of-function phenotype in BMP signaling. Among these, previously identified components, including the two BMP receptors Put and Tkv, and Mad, were top candidates. Furthermore, previous studies have reported that several other candidates, including nejire (nej)/CREB-binding protein (CBP), Daxx-like protein (DLP), shaggy (sgg)/Glycogen synthase kinase3 (GSK3), or MAN1/LEM domain-containing protein, interact genetically with the BMP signaling pathway [19]–[23]. These findings suggest that our RNAi screening is a useful strategy to identify novel components in signaling pathways. The hit list also contains several hitherto uncharacterized genes that remain to be addressed. In the present study, one of the hits, CG2331/Ter94, a member of the family of AAA ATPases, was selected for further analysis.

**Table 1:** Candidate genes involved in BMP signaling in *Drosophila* S2 cells.

Rank*	SampleID	DGC cloneID	Annotation ID(CG number)	Symbol	Humanortholog	Molecular structure/function
RNAi phenotype: loss-of-BMP signaling						
1	200	LD31893	CG7904	put	BMP-RII	TGF-β type II receptor/protein kinase
2	383	RE72705	CG12399	Mad	Smad1/5/8	SMAD domain/protein binding
3	140	GH25238	CG14026	tkv	BMP-RI	TGF-β type I receptor/protein kinase
4	199	LD33277	CG15319	nej	CREBBP	Zinc finger/histoneacetyltransferase
5	62	LD35644	CG1057	MED31	MED31	Mediator complexsubunit/transcription coactivator
6	47	LP12034	CG2331	Ter94	VCP/p97	AAA+ ATPasedomain/CDC48 family
7	124	SD07852	CG4214	Syx5	STX5	SNARE coiled-coildomain/SNAP receptor activity
8	197	LD31537	CG9537	DLP	DAXX	Daxx protein/protein binding
9	225	AT07244	CG2331	Ter94	VCP/p97	AAA+ ATPasedomain/CDC48 family
10	134	LD45157	CG4722	bib	unknown	Major intrinsicprotein/cation channel activity
11	25	LP03545	CG7031	CG7031	unknown	unknown
12	229	AT24649	CG14472	poe	UBR4	Zinc finger/ubiquitinligase activity
13	233	GM02885	CG2331	Ter94	VCP/p97	AAA+ ATPasedomain/CDC48 family
14	111	LD44595	CG2621	sgg	GSK3	Ser/Thr protein kinase activity
15	29	LP08442	CG3546	CG3546	unknown	unknown
16	35	SD17630	CG42788	CG42788	FRMPD	FERM domain, PDZ domain
17	243	GM09915	CG15611	CG15611	unknown	Dbl homology domain
18	102	LD27620	CG5644	CG5466	unknown	unknown
RNAi phenotype: gain-of-BMP signaling						
	437	RE60089	CG3167	MAN1	LEMD3	LEM domain

*Rank shows the order of loss-of-BMP signaling due to RNAi.

### Ter94 is a novel component that regulates BMP signaling in *Drosophila* cells

**Figure 2:**
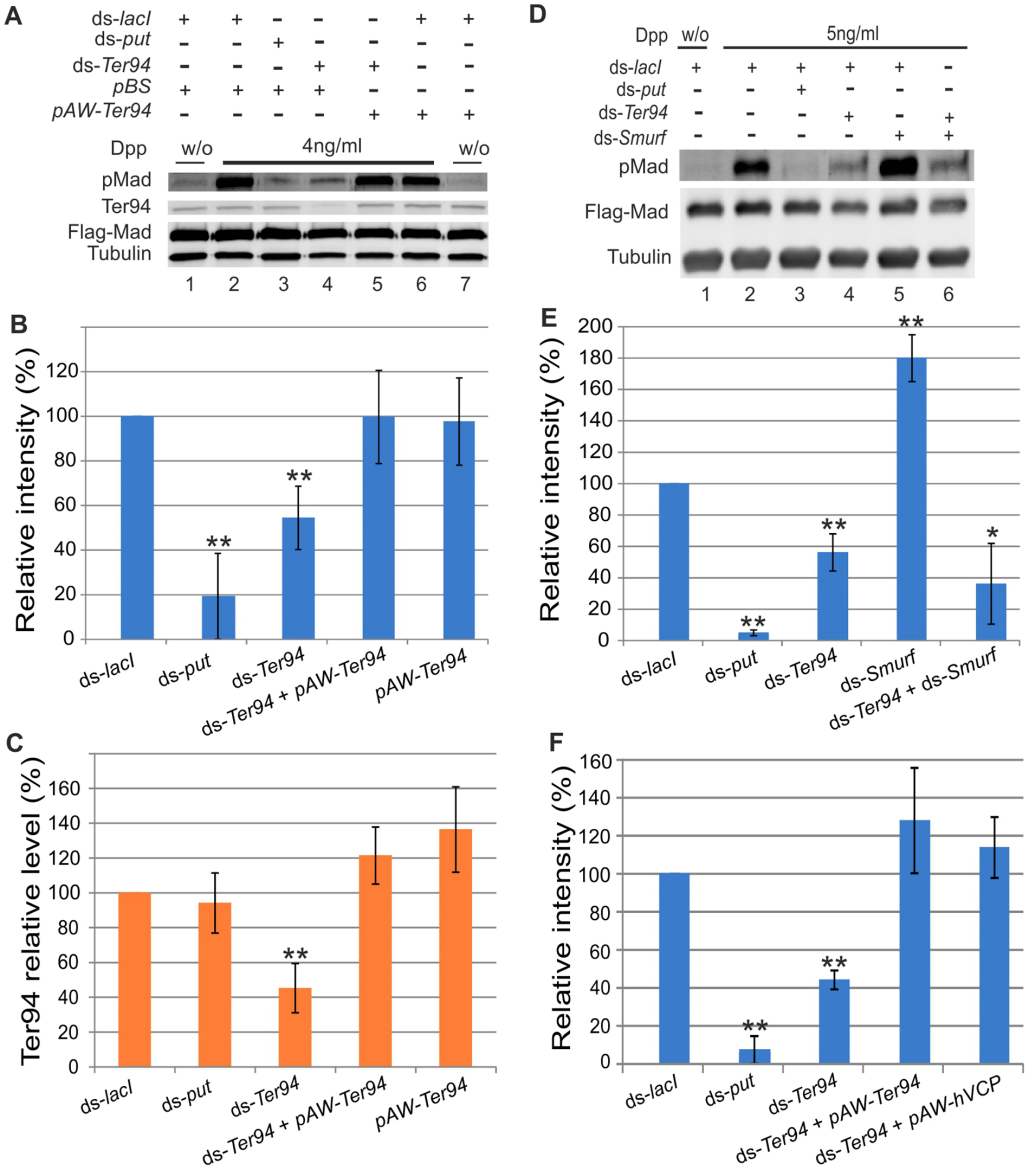
Ter94 is involved in BMP signaling in *Drosophila* S2 cells. (A) BMP signals are regulated by Ter94 in S2 cells. The loss of BMP signaling caused by *Ter94* (3′-UTR) RNAi was restored by the ectopic expression of *Ter94*. *pAW-Ter94* was used for the expression of *Ter94* in S2 cells; whereas *pBS* served as a control vector. Tubulin was used for a loading control. (B) The relative intensities of pMad signals (compared to the pMad signal of ds-*lacI*) were measured with Western blotting. pMad signals were normalized by Tubulin expression; ds-*lacI* intensity was set at 100%. ** indicates P<0.01. (C) The relative protein level of Ter94 (compared to the protein level of ds-*lacI-*treated cells) was quantified. The Ter94 level was normalized by Tublin expression; ds-*lacI* intensity was set at 100%. ** indicates P<0.01. (D) Ter94 is involved in BMP signaling upstream of Smurf or independent of Smurf in S2 cells. Tubulin was used for a loading control. (E) The relative intensities of pMad signaling (compared to the pMad signal of ds-*lacI*) were measured with Western blotting. pMad signals were normalized by Tubulin expression; ds-*lacI* intensity was set at 100%. * indicates P<0.05 and ** indicates P<0.01. Note that the pMad signal of the double knockdown of *Ter94* and *Smurf* is similar to that of *Ter94* RNAi. (F) The loss of BMP signaling caused by *Ter94* RNAi was restored by the ectopic expression of *hVCP*. The relative intensities of pMad signaling (compared to the pMad signal of ds-*lacI*) were measured with Western blotting. *pAW-Ter94* or *pAW-hVCP* was used for the expression of *Ter94* or *hVCP* in S2 cells, respectively. pMad signals were normalized by *Flag-Mad* expression; ds-*lacI* intensity was set at 100%. ** indicates P<0.01. dsRNA against *lacI* served as a control to rule out any specific effects caused by RNAi transfection, and *put* was used to validate that its knockdown inhibits pMad signals.

One of the top candidates in the RNAi screening was CG2331/Ter94 (Table 1). Ter94 is an ortholog of Cdc48/valosin-containing protein (VCP) which has been shown to be involved in various biological functions [24], [25]. To confirm that Ter94 is involved in the BMP signaling pathway in S2 cells, we studied whether loss of BMP signaling by *Ter94* RNAi could be rescued by ectopic expression of *Ter94*. For this purpose, we performed Western blotting using pMad antibody to evaluate BMP signaling in S2 cells. Exogenously adding Dpp to S2 cells that overexpress Flag-Mad induced a pMad signal (Fig. 2A, lane 2), which was significantly reduced by the knockdown of either *Ter94* or *put* (Figs. 2A (lanes 3, 4), B). The BMP signal was efficiently recovered when dsRNA against *Ter94* 3′UTR was co-transfected with a *Ter94* expression plasmid in S2 cells (Figs. 2A (lane 5), B). We noted that the transfection of S2 cells with a pAW-*Ter94* expression plasmid led to no additional enhancement of BMP signaling (Figs. 2A (lane 6), B). This is not surprising, since Ter94 is already expressed at high levels endogenously, and ectopic expression only slightly elevates total Ter94 levels (Fig. 2C). These results suggest that Ter94 is required for BMP signaling in S2 cells.

Since Ter94 ortholog VCP in vertebrates have been shown to be involved in the ubiquitin-proteosome pathway [24], [25], we wondered whether Ter94 regulates BMP signaling in this manner. It has been shown that the ubiquitin E3 ligase Smurf downregulates BMP signals through the ubiquitin-mediated degradation of Mad proteins [26], [27]. In *Drosophila* S2 cells, the knockdown of *Smurf* upregulates BMP signaling (Figs. 2D (lane 5), E). Knocking down both *Ter94* and *Smurf* reduced Mad phosphorylation, which resembled the one caused by *Ter94* RNAi alone (Figs. 2D (lanes 4, 6), E). These data indicate that Ter94 is involved in regulating BMP signaling upstream of Mad phosphorylation or by repressing pMad turnover independent of Smurf proteins.

### VCP is a component that regulates BMP signaling in mammalian cells

**Figure 3:**
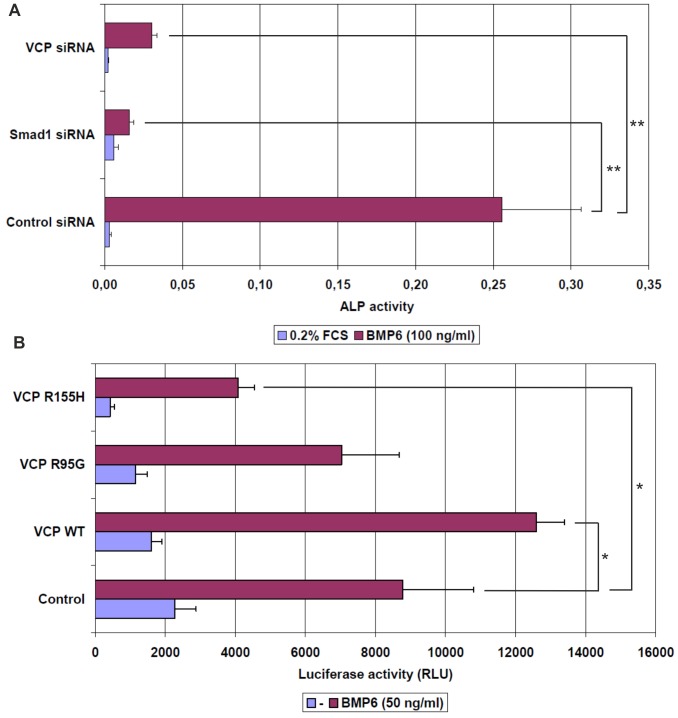
VCP is involved in BMP signaling in mammalian cells. (A) Alkaline phosphatase (ALP) assay in C2C12 cells. BMP6-induced ALP activity was abolished by the knockdown of Smad1 or VCP. ** indicates P<0.01. (B) BMP-induced activity of the luciferase reporter (BRE-Luc) in KS483 cells. BMP6-induced luciferase activity was inhibited by the overexpression of VCP^R155H^ but was activated by the overexpression of VCP^WT^. * indicates P<0.05.

We then wondered whether Ter94 is required for BMP signaling in species other than *Drosophila*. BMP family signaling molecules are highly conserved between vertebrates and *Drosophila* [2]. Ter94 is an ortholog of the VCP/Cdc48 family, which is highly conserved from yeast to human [24], [25]. First, we studied whether mammalian VCP can compensate for the loss of *Drosophila* Ter94 in S2 cells. The *Ter94* RNAi phenotypes were efficiently rescued by human VCP (hVCP) as well as Ter94 in S2 cells (Fig. 2F), suggesting that hVCP retains the ability to regulate BMP signaling even in *Drosophila* cells. We then investigated whether VCP is involved in BMP signaling in mammalian cells. When mouse premyoblast C2C12 cells [17] were incubated with BMP6 alkaline phosphatase (ALP) activity was induced (Fig. 3A). In contrast, the knockdown of VCP or Smad1 by siRNA significant reduced ALP activity (Fig. 3A). We further tested VCP function in luciferase reporter assays by measuring BMP-induced luciferase reporter (BRE-Luc) activity in mouse pluripotent mesenchymal KS483 cells [17] overexpressing either two missense mutations, VCP^R95G^ and VCP^R155H^, responsible for Inclusion body myopathy associated with Paget disease of the bone and frontotemporal dementia (IBMPFD), or wild-type VCP [28], [29]. Wild-type VCP induced higher luciferase activity than control; in contrast, one of the mutant VCP^R155H^, when overexpressed, reduced luciferase activity (Fig. 3B), indicating that VCP^R155H^ may have a dominant negative effect against BMP signaling. Taken together, these results suggest that Ter94/VCP is an evolutionarily conserved regulator of BMP signaling.

### Ter94 is involved in dorsoventral patterning of the blastoderm embryo through regulating BMP signaling

**Figure 4:**
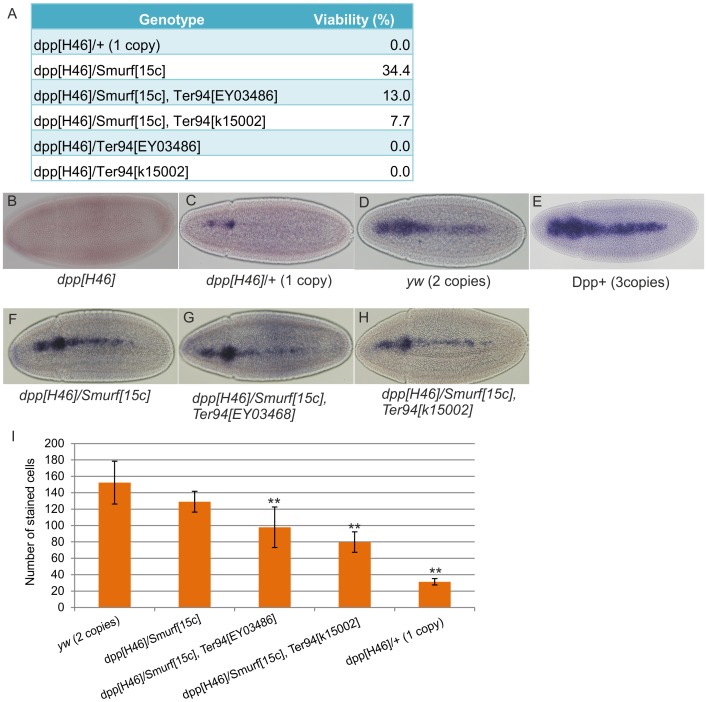
Ter94 is involved in BMP signaling *in vivo*. (A) *Ter94* alleles and *dpp* show genetic interactions. The *dpp* null mutant is haploinsufficient. In a *Smurf* mutant background, the *dpp* heterozygote is partially viable. In *Ter94* and *Smurf* double mutants, the viability of the *dpp* heterozygote is less than that of the *Smurf* mutant alone. (B–H) A dorsal view of *race* mRNA in early stage-6 embryos. *dpp* null (B), *dpp* heterozygote (C), wild-type phenotype (D), three copies of Dpp (+/P[*dpp*-P23]: E), transheterozygous for *dpp* null and *Smurf* mutant alleles (*dpp^H46^ Smurf^+^/dpp^+^ Smurf^C15^*: F), transheteorzygous for *dpp* null, *Smurf* mutant and *Ter94* mutant alleles (*dpp^H46^ Ter94^+^ Smurf^+^/dpp^+^ Ter94^EY03468^ Smurf^C15^*: G, *dpp^H46^ Ter94^+^ Smurf^+^/dpp^+^ Ter94^k15002^ Smurf^C15^*: H). (I) Number of *race* staining cells in early stage-6 embryos. ** indicates *P*<0.01 (compared with wild-type phenotype *yw*).

To understand how Ter94 mediates BMP signaling *in vivo*, we studied various alleles of *Ter94* in *Drosophila*. During early embryogenesis, BMP morphogen gradient formation is crucial for dorsal patterning of the *Drosophila* embryo [12], [30]. Previous studies have shown that Ter94 is involved in oogenesis and that loss of *Ter94* leads to defects in oocyte development. Consequently, embryos from germline clones of *Ter94* mutants are unavailable [14], [31]. We therefore investigated genetic interactions between *Ter94* and *Drosophila* BMP ligand *dpp*, which is known to be haploinsufficient; moreover, graded activities of the BMP signaling in the early embryo is *dpp*-dosage sensitive [5], [32]. Flies transheterozygous for *Smurf^15C^* and *dpp* null allele (*dpp^H46^*) are viable, since *Smurf* mutations cause a spatial increase in BMP signaling (34.4% viable, n = 221) (Fig. 4A) [26]. By producing double mutants of *Ter94* and *Smurf*, we examined genetic interactions between *Ter94* and *dpp*. Flies transheterozygous for *Smurf^15C^-Ter94^K15002^* and *dpp^H46^*, or *Smurf^15C^-Ter94^EY03486^* and *dpp^H46^* are less viable (7.7%, n = 209 or 13.0% n = 231, respectively) (Fig. 4A). These data indicate that *Ter94* and *dpp* have genetic interactions in *Drosophila* development.

To understand how the BMP signal is regulated by Ter94 during embryo development, we studied the expression of a BMP target gene in the embryos. The *race* gene is expressed at the dorsal-most cells regulated by high-level BMP signaling during the blastoderm stage [18]. Since *race* expression occurs in a Dpp dose-dependent manner (Figs. 4B–E), we studied *race* expression in the embryo transheterozygous for *Smurf, Ter94* and *dpp*. *race* expression is only detected at the head part in one copy of *dpp* (heterozygotes for *dpp^H46^*) (Figs. 4C, I). In contrast, flies transheterozygous for *Smurf* and *dpp* show a similar pattern to that of wild-type embryos (Fig. 4F, I). The numbers of *race*-expressing cells decreased in flies tranheterozygous for *Smurf, Ter94* and *dpp* (Figs. 4G–I). These data suggest that Ter94 regulates BMP signaling during *Drosophila* embryo development. The data are also consistent with the fact that Ter94 helps maintaining viability under sensitized conditions of BMP signaling.

We further studied genetic interactions between *Mad*, *Ter94* and *dpp,* or *tkv, Ter94* and *dpp* by using *Mad, Ter94* or *tkv, Ter94* double mutants. Flies transheterozygous for *Mad^12^* and hypomorphic allele *dpp^hr4^* showed low viability (Table 2), whereas, on this sensitized background, flies transheterozygous for *Mad*, *Ter94* and *dpp* were non-viable. Moreover, *Ter94* showed strong genetic interactions with BMP type I receptor *tkv,* since flies transheterozygous for *tkv, Ter94* and *dpp^hr4^* showed significantly reduced viability.

**Table 2:** Genetic interactions between Ter94 and the BMP signals.

Genotype	Viability (%)
dpp[hr4]/Mad [12]	16.4
dpp[hr4]/Mad [12], Ter94[26]-[8]	0
dpp[hr4]/Mad [12], Ter94[22]–[30]	0
dpp[hr4]/Mad [12], Ter94 [03775]	0
dpp[hr4]/Mad [12], Ter94[k15002]	0
dpp[hr4]/tkv [8]	120.0
dpp[hr4]/tkv [8], Ter94[26]-[8]	11.4
dpp[hr4]/Ter94[26]-[8]	91.0
dpp[hr4]/Ter94[22]–[30]	73.5
dpp[hr4]/Ter94 [03775]	101.1
dpp[hr4]/Ter94[k15002]	97.9

Taken together, these results indicate the role of Ter94 in regulating BMP signaling *in vivo*.

## Discussion

In this work, we established a high throughput RNAi screening in *Drosophila* S2 cells to identify novel components involved in BMP signaling. One of the top hits was Ter94, an ortholog of Cdc48/VCP. Our data reveal that Ter94 and VCP are required for BMP signaling in *Drosophila* S2 cells and mammalian cells, respectively. We also found that Ter94 is required for BMP signaling during *Drosophila* embryogenesis.

### RNAi screening

The development of RNAi technology advanced our approach to investigating a loss-of-function analysis of gene functions. Applying a high throughput RNAi approach allowed us to perform genome-wide screening to identify novel components of signal transduction and cell responses to environmental stimuli in cell culture [11]. Although TGF-β type signal transduction has been intensely studied in recent decades, components that mediate these signaling pathways remain to be discovered. Recent genome-wide RNAi screening that explores the factors influencing the localization of *Drosophila* Mad in S2 cells identified pyruvate dehydrogenase phosphatase (PDP) and nuclear membrane bound protein moleskin (msk) as novel regulators of BMP signaling [33], [34]. In this study, a screening system has been established, by detecting phosphorylated Mad localized in the nucleus as a readout of BMP signals and applied to the genome-wide approach. Screening revealed the involvement of 17 genes in BMP signaling in *Drosophila* S2 cells. These genes contain essential components of the canonical BMP pathway, including Tkv, Punt, and Mad as well as modulators of BMP signal transduction, including nej/CBP, DLP, sgg/GSK3 and MAN1 (Table 1). These results indicate that our screening system is useful for identifying components that are involved in BMP signaling in tissue culture cells. Importantly, our screening system also identified several uncharacterized genes, including bib, CG7031, CG3546, CG42788, CG15611 and CG5466 (Table 1). The molecular mechanisms of these genes in BMP signal transduction remain to be addressed in the future.

### Ter94/VCP and BMP signaling

Ter94 is considered a functional ortholog of Cdc48 in budding yeast, as is VCP in mammals; both have been thoroughly studied. They are chaperone-like ATPases that control the degree of ubiquitination of bound substrates [24], [25]. In *Drosophila* several *Ter94* mutant alleles have been isolated, some of which are strong alleles showing incomplete oogenesis in germline clones [14], [31]. *Drosophila* Ter94 has been widely studied as a disease model of inclusion body myopathy with early-onset Paget disease and frontotemporal dementia (IBMPFD) [35], [36], a model of interaction with retinal pathology caused by misfolded rhodopsin [37], and one of interaction with polyglutamine (polyQ) disease caused by expression of expanded polyQ [38]. This study provides evidence that Ter94/VCP appear to play a conserved role as a regulator of BMP signaling. Recent studies also suggest that VCP may play a role in BMP signaling during spermatogenesis, since Smad1, pSmad1 and VCP are co-localized in the postnatal rat testes and epididymis [39]. Although the molecular mechanisms of how Ter94/VCP regulates BMP signal transduction remain to be addressed, Ter94 is likely to function upstream of the phosphorylation of Mad, such as the regulation of stability or localization of receptors, or to regulate pMad stability independent of Smurf.

Since Ter94/VCP is involved in various biological processes, investigating the contributions of Ter94/VCP to BMP signaling *in vivo* was challenging. Indeed, mutant clones of *Ter94* in the wing imaginal discs were eliminated during larvae stages and could not be analyzed for BMP signaling (data not shown). This study revealed strong genetic interaction of *Ter94* with BMP signaling pathways, including BMP ligand *dpp*, *Mad* or BMP type I receptor *tkv* in sensitized conditions. Moreover, Ter94 is required for maintaining BMP signaling during early embryogenesis. Since the BMP activity gradient needed for dorsal patterning in the *Drosophila* blastoderm embryo is maintained through the feedback circuit [40], Ter94 may play a role in the positive feedback loop.

In summary, this study shows that Ter94 andVCP play conserved roles in modulating BMP signals. Further studies will address the molecular mechanisms underlying how Ter94 andVCP regulate BMP signaling and the *in vivo* functions through which Ter94/VCP modulates BMP signals.

## Supporting Information

Table S1 Candidates that are involved in BMP signaling after primary screen.
(PDF)

Table S2 Candidate genes that are involved in BMP signaling after secondary screen.
(PDF)
