# Supplementary material for: Ter94/VCP Is a Novel Component Involved in BMP Signaling
Source: PLoS One. 2014 Dec 3;9(12):e114475. doi: 10.1371/journal.pone.0114475 (PMC4255028; doi:10.1371/journal.pone.0114475)
Supplement: Table S1 — Candidates that are involved in BMP signaling after primary screen. (PDF) [file pone.0114475.s001.pdf]

Table S1. Candidates that are involved in BMP signaling after primary screen

Sample ID	DGC clone ID	Annotation ID (CG number)	Symbol
1	GH10049	CG8285	boss
2	GH08336	CG31207	CG31207
3	GH07711	CG5851	sds22
4	GH05104	CG13607	CG13607
5	GH06563	CG7073	Sar1
6	GH11073	CG2267, CG10305	CG2267, RpS26
7	GH14362	CG11727	CG11727
8	GH19261	CG14782	rush
9	GH16343	CG8507	CG8507
10	GH13168	CG5778	CG5778
11	GH17513	CG11700	CG11700
12	GH21935	CG5179	Cdk9
13	GH24939	CG17108	CG17108
14	GM19936	CG18591	SmE
15	GM26647	CG4464	RpS19a
16	GM27203	CG6163, CG11271	CG6163, RpS12
17	GM30781	CG1771	mew
18	HL07910	CG10077	CG10077
19	GM23968	CG16792	SmF
20	LD46784	CG9696, CG1939	dom,Dpck
21	LD34822	CG1424	mst
22	LD47488	CG6779	RpS3
23	LD37859	CG10423	RpS27
24	LD40873	CG7434	RpL22
25	LP03545	CG7031	CG7031
26	LP03403	CG3880, CG8053	CG3880, eIF-1A
27	LP06778	CG5650	Pp1-87B
28	LP06330	CG32638, CG11276	CG32638, RpS4
29	LP08442	CG3546	CG3546
30	LP11750	CG6041	CG6041
31	LP10071	CG1873, CG8280	EF1α100E, Ef1α48D
32	SD08670	CG13628	Rpb10

33	SD12691	CG11276	RpS4
34	LP12057	CG16916	Rpt3
35	SD17630	CG42788	CG42788
36	SD17528	CG9280	Glt
37	SD17959	CG3064	futsch
38	SD19967	CG14721	CG14721
39	SD22208	CG4918	RpLP2
40	SD24044	CG5057	MED10
41	SD24632	CG4496	CG4496
42	LD24589	CG17521	Qm
43	LD25644	CG9264	CG9264
44	LD34409	CG5320	Gdh
45	GH27257	CG12076	YT521-B
46	LD32685	CG15817	CG15817
47	LP12034	CG2331	TER94
48	SD11109	CG17800	Dscam
49	SD21550	CG13204	CG13204
50	LD33831	CG10837	eIF-4B
51	LP23833	CG10514	CG10514
52	LP19917	CG3131	CG3131
53	GH12596	CG3051	SNF1A
54	GH11973	CG6014	CG6014
55	LD26050	CG12340	CG12340
56	LD26519	CG11371	dbr
57	GH10833	CG5045	CG5045
58	GH15335	CG12284	th
59	GH19356	CG13631	CG13631
60	GH21964	CG11546	l(2)02045
61	GH18603	CG6233	Ufd1-like
62	LD35644	CG1057	MED31
63	LD35705	CG7281	CycC
64	LD36125	CG4454	Borr
65	LD36256	CG17358	Taf12
66	LD46714	CG7081	CG7081
67	LP10918	CG6467	Jon65Aiv
68	LP02988	CG4471	Tsp42Ep

69	SD08771	CG9053	CG9053
70	SD02332	CG18495	Prosalpha6
71	LP05929	CG9675	CG9675
72	SD10560	CG17183	MED30
73	GH20776	CG8416	Rho1
74	GM13047	CG8922	RpS5a
75	GM13889	CG10161	eIF-3p66
76	LD38389	CG1519	Prosalpha7
77	LD38718	CG5363	cdc2
78	LD46084	CG8609	Trap36
79	LP01553	CG5431	CG5431
80	LD21701	CG3193	crn
81	LD22387	CG3180	RpII140
82	LD22754	CG2925	noi
83	LD23808	CG11271	RpS12
84	LD22701	CG8264	Bx42
85	LD23157	CG5605	eRF1
86	LD23532	CG16817	CG16817
87	LD23674	CG2998	RpS28b
88	LD21601	CG6625	Snap
89	LD21741	CG5537	CG5537
90	LD22648	CG11888	Rpn2
91	LD23744	CG7173	CG7173
92	LD23958	CG13389	RpS13
93	GH01724	CG11139	p47
94	GH02029	CG4894	Ca-alpha1D
95	GH03876	CG18802	alpha-Man-II
96	GH07782	CG9747	CG9747
97	GH04632	CG32245	CG32245
98	GH09594	CG12582	CG12582
99	LD29131	CG7831	ncd
100	LD29218	CG6197	CG6197
101	LD27504	CG15669	MESK2
102	LD27620	CG5466	CG5466
103	LD30122	CG5014	Vap-33-1
104	LD36410	CG11798	chn

105	LD39166	CG4451	Hs6st
106	LD35441	CG2139	aralar1
107	LD38742	CG6506	CG6506
108	LD38749	CG30497	CG30497
109	LD39266	CG7073	sar1
110	LD39624	CG3209	CG3209
111	LD44595	CG2621	sgg
112	LD43674	CG1420	CG1420
113	SD10366	CG10701	Moe
114	SD08021	CG9556	alien
115	GH12111	CG9696	dom
116	GH24002	CG3331	e
117	GM14109	CG9075	eIF-4a
118	LD23644	CG7269	Hel25E
119	LD36178	CG6975	gig
120	LD32416	CG1404	ran
121	SD07148	CG1341	Rpt1
122	LP01188	CG12104	CG12104
123	LD45152	CG3605	CG3605
124	SD07852	CG4214	Syx5
125	LD48007	CG1710	Hcf
126	LD24894	CG3618	CG3618
127	GH14582	CG7776	E(Pc)
128	GH16721	CG6376	E2f
129	GH18004	CG11064	RfaBp
130	GH23825	CG11448	CG11448
131	LD29902	CG7913	PP2A-B'
132	LD31045	CG3445	phol
133	LD31286	CG10944	RpS6
134	LD45157	CG4722	bib
135	LD44234	CG3329	Prosbeta2
136	LD45403	CG10084	CG10084
137	SD02026	CG5055	baz
138	SD04748	CG4062	Aats-val
139	SD04170	CG11033	CG11033
140	GH25238	CG14026	tkv

141	LD34489	CG30420	CG30420
142	SD03723	CG10805	CG10805
143	SD10782	CG11661	Nc73EF
144	LD33331	CG7837	CG7837
145	GH23735	CG8205	fus
146	LD30271	CG15010	ago
147	LD26789	CG12234	Ranbp21
148	LD24159	CG4097	Pros26
149	GM13041	CG3817	CG3817
150	GH01072	CG6884	Med21
151	GH25878	CG7913	PP2A-B'
152	GM15484	CG14792	sta
153	LP01382	CG4204, CG7808	EloB, RpS8
154	LP02768	CG8717	slv
155	LP04961	CG33113	Rtnl1
156	GM14481	CG11207	feo
157	LP12144	CG14206	RpS10b
158	SD20666	CG6223	betaCop
159	SD19962	CG1973	CG1973
160	SD27452	CG8727	cyc
161	GH17801	CG17660	CG17660
162	LD46954	CG8370	CG8370
163	GH01429	CG7379	CG7379
164	LD24895	CG3167	CG3167
165	GH12023	CG10286	CG10286
166	GH05039	CG2096	flw
167	GH14384	CG9131	slmo
168	GH01077	CG3731	CG3731
169	GH14674	CG33084	DNApol-γ35
170	GH10215	CG8772	nemy
171	GH13134	CG9261	nrv2
172	GH16485	CG12191	dpr20
173	GH01107	CG1725	dlg1
174	GH14162	CG11910	alrm
175	GH14380	CG12239	CG12239
176	GH09663	CG16833	CG16833

177	GH09884	CG30384	CG30384
178	GH10478	CG15309	CG15309
179	GH16161	CG15279	CG15279
180	GH16625	CG9166	312,0
181	GH17632	CG10824	CG10824
182	GH17801	CG17660	CG17660
183	GH18014	CG1600	Drat
184	GH24286	CG9663	CG9663
185	LD43558	CG1554	RpII215
186	LD44381	CG15532	hdc
187	SD02490	CG12031	MED14
188	LD38671	CG5634	dsd
189	LD25641	CG7282	CG7282
190	SD03094	CG9311	CG9311
191	LD46954	CG8370	CG8370
192	GH21134	CG6741	arc
193	SD04853	CG17233	CG17233
194	GH01409	CG5166	Atx2
195	GH11341	CG1100	Rpn5
196	GH02649	CG7366	CG7366
197	LD31537	CG9537	DLP
198	LD32255	CG17610	grk
199	LD33277	CG15319	nej
200	LD31893	CG7904	put
201	LD33976	CG10772	Fur1
202	GH12584	CG6438	amon
203	GH01053	CG1168	7B2
204	LD30182	CG18734	Fur2
205	AT09438	CG4878	eIF3-S9
206	AT07769	CG7542	CG7542
207	AT07692	CG31501	nxf4
208	AT13263	CG7003	CG7003
209	AT13773	CG5207	scpr-A
210	AT13932	CG33142	CG33142
211	AT25373	CG31287, CG17654	CG31287, Eno
212	AT24439	CG7229	CG7229

213	AT02695	CG2171	Tpi
214	AT04751	CG12289	CG12289
215	AT05318	CG32319	CG32319
216	AT22129	CG15532	hdc
217	AT13329	CG8495	RpS29
218	AT17601	CG1519	Prosalpha7
219	AT28563	CG8495	RpS29
220	AT25963	CG5977	spas
221	AT27578	CG9412	rin
222	AT30656	CG5983	ACXC
223	AT23070	CG15515	CG15515
224	AT03646	CG6292	CycT
225	AT07244	CG2331	TER94
226	AT07973	CG3416	Mov34
227	AT15146	CG10484	Dox-A2
228	AT20865	CG11624	Ubi-p63E
229	AT24649	CG14472	poe
230	LD14119	CG3479	osp
231	LD13852	CG8815	Sin3A
232	LD20667	CG11376	CG11376
233	GM02885	CG2331	TER94
234	LD06574	CG5183	KdelR
235	LD09978	CG7885	RpII33
236	LD11847	CG12324	RpS15Ab
237	LD12894	CG4993	PRL-1
238	GM04108	CG7292	Rrp6
239	LD21089	CG8367	cg
240	GM05133	CG32555	RhoGAPp190
241	HL04250	CG6450	lva
242	GM09620	CG5931	CG5931
243	GM09915	CG15611	CG15611
244	GM01240	CG13900	CG13900
245	GM02347	CG17608	fu12
246	LD02553	CG6064	TORC
247	LD08906	CG4936	CG4936
248	LD16029	CG6120	Tsp96F

249	LD19039	CG32000	CG32000
250	LD08432	CG10278	GATAe
251	LD13191	CG17233	CG17233
252	LD08231	CG45049	CG45049
253	LD11409	CG9940	CG9940
254	LD12453	CG32904	seq
255	LD07466	CG5634	dsd
256	HL01707	CG7762	Rpn1
257	LD09376	CG14792	sta
258	LD11482	CG3395	RpS9
259	GM06171	CG7977	RpL23A
260	LD11783	CG31811	cenG1A
261	LD10287	CG12238	e(y)3
262	LD14049	CG5352	SmB
263	GM03914	CG7524	Src64B
264	GM04767	CG11180	CG11180
265	GM03174	CG11246	Rpb8
266	GM05057	CG7382	CG7382
267	GM06171	CG7977	RpL23L
268	GM03014	CG5903	CG5903
269	LD05365	CG12314	CG12314
270	LD05688	CG5185	Tom
271	GM04029	CG11488	mRpL10
272	LD18389	CG6171	CG6171
273	LD16326	CG2746	RpL19
274	HL02010	CG4396	fne
275	GM02242	CG3203	RpL17
276	LD17235	CG7726	RpL11
277	LD07883	CG10375	CG10375
278	GM10051	CG10811	eIF-4G
279	GM10122	CG10215	Ercc1
280	LD01958	CG4330	CG4330
281	GM01793	CG11523	CG11523
282	GM01970	CG4043	Rrp46
283	GM02143	CG14804	CG14804
284	GM09283	CG14715	CG14715

285	HL02449	CG7313	CheA75a
286	LD05303	CG32000	CG32000
287	LD02192	CG4570	CG4570
288	LD03052	CG5445	CG5445
289	LD03419	CG8416	Rho1
290	GM01519	CG9248	CG9248
291	LD15851	CG9553	chic
292	LD08529	CG7938	Sry-β
293	LD07688	CG12254	MED25
294	LD11064	CG1664	sbr
295	LD09689	CG15845	Adf1
296	LD10456	CG3278	Tif-IA
297	LD14353	CG1793	MED26
298	LD14913	CG8073	Pmm45A
299	LD15209	CG3918	CG3918
300	LD16579	CG17104	CG17104
301	LD13525	CG14162	dpr6
302	LD03241	CG11430	olf-186
303	RE01736	CG7890	Hs3st-B
304	RE01910	CG32796	CG32796
305	RE01528	CG4563	CG4563
306	RE03692	CG5582	CG5582
307	RE04143	CG6198	CHORD
308	RE04220	CG2168	RpS3A
309	RE04676	CG31265	CG31265
310	RE05370	CG9194	CG9194
311	RE05287	CG11125	CG11125
312	RE05093	CG11765	Prx2540-2
313	RE05533	CG13743	CG13743
314	RE04130	CG15739	CG15739
315	RE09339	CG9358	Phk-3
316	RE06857	CG4742	mRpL22
317	RE10012	CG4158	wor
318	RE11206	CG18657	NetA
319	RE12054	CG3287	CG3287
320	RE13143	CG10371	CG10371

321	RE09982	CG9503	CG9503
322	RE12569	CG10924	CG10924
323	RE16081	CG40222	Rad21
324	RE16426	CG6403	CG6403
325	RE16694	CG5883	CG5883
326	RE18374	CG31005	CG31005
327	RE18653	CG1883	RpS7
328	RE19835	CG5800	CG5800
329	RE20268	CG2051	CG2051
330	RE20544	CG31243	cpo
331	RE20784	CG15743	CG15743
332	RE22207	CG11218	Obp56d
333	RE23388	CG3455	Rpt4
334	RE26382	CG6510	RpL18A
335	RE26538	CG5849	CG5849
336	RE26559	CG14780	CG14780
337	RE26656	CG1153	Osi7
338	RE26840	CG33108	CG33108
339	RE26924	CG13969	bwa
340	RE26983	CG18013	CG18013
341	RE27528	CG9253	CG9253
342	RE27841	CG3267	CG3267
343	RE28911	CG45090, CG45091	SclA, SclB
344	RE29270	CG4153	eIF-2beta
345	RE33778	CG17508	CG17508
346	RE35481	CG31102	CG31102
347	RE37682	CG7785	CG7785
348	RE39081	CG8254	exex
349	RE39106	CG10406	mRpS33
350	RE41410	CG7264	CG7264
351	RE42721	CG11377	CG11377
352	RE43020	CG12017	CG12017
353	RE44811	CG3753	Marcal1
354	RE44251	CG14117	CG14117
355	RE45003	CG14500	CG14500
356	RE45077	CG2772	CG2772

357	RE45331	CG43369	Mitf
358	RE48939	CG3780	Spx
359	RE53354	CG11027	Arf102F
360	RE53486	CG4756	CG4756
361	RE54483	CG2033	RpS15Aa
362	RE54849	CG8053	eIF-1A
363	RE55472	CG12265	CG12265
364	RE55814	CG6662	CG6662
365	RE56731	CG12758	sano
366	RE55111	CG9922	CG9922
367	RE66843	CG3320	Rab1
368	RE67616	CG15173	CG15173
369	RE68460	CG3935	al
370	RE68515	CG14543	CG14543
371	RE68984	CG1873	Ef1alpha100E
372	RE69372	CG5333	trus
373	RE70632	CG1810	mRNA-cap
374	RE71127	CG17633	CG17633
375	RE71708	CG5677	Spase22-23
376	RE72594	CG31762	aret
377	RE03680	CG8332	RpS15
378	RE08425	CG7283	RpL10Ab
379	RE17389	CG7239	CG7239
380	RE44901	CG8392	l(2)05070
381	RE69521	CG2163	Pabp2
382	RE72235	CG17521	Qm
383	RE72705	CG12399	Mad
384	RE75106	CG11357	CG11357
385	RH04426	CG4264	Hsc70-4
386	RH04612	CG1524	RpS14a
387	RH04903	CG4897	RpL7
388	RH06886	CG7808	RpS8
389	RH06643	CG8495	RpS29
390	RH06581	CG8900	RpS18
391	RH07244	CG7808	RpS8
392	RH07540	CG4046	RpS16

393	RH08962	CG15697	RpS30
394	RH08058	CG7322	CG7322
395	RH09938	CG10652	RpL30
396	RH12258	CG6619	CG6619
397	RH20790	CG8495	RpS29
398	RH21245	CG5887	desat1
399	RH25914	CG1524	RpS14a
400	RH25462	CG31775	CG31775
401	RH33355	CG10979	CG10979
402	RH35331	CG1810	mRNA-cap
403	RH32624	CG13222	CG13222
404	RH33439	CG6846	RpL26
405	RH34416	CG13779	Sem1
406	RH35480	CG7977	RpL23A
407	RH35475	CG9742	CG9742
408	RH40396	CG6707	CG6707
409	RH42089	CG7808	RpS8
410	RH41593	CG5827	RpL37A
411	RH39663	CG1263	RpL8
412	RH40310	CG2960	RpL40
413	RH48056	CG9354	RpL34b
414	RH47995	CG15693	RpS20
415	RH53853	CG1793	Arc70
416	RH56796	CG7137	CG7137
417	RH62830	CR33946	pncr008:3L
418	RH61467	CG12772	CG12772
419	RH66170	CG5258	NHP2
420	RH69713	CG16799	CG16799
421	RH68811	CG17364	CG17364
422	RH69856	CG14880	CG14880
423	RH69586	CG11765	Prx2540-2
424	RH72196	CG17331	CG17331
425	RE22983	CG5410	CG5410
426	RE06042	CG7283	RpL10Ab
427	RH44168	CG13737	CG13737
428	RE29621	CG11888	Rpn2

429	RE31802	CG4454	CG4454
430	RE38584	CG4861	LpR1
431	RE44119	CG3922	RpS17
432	RE44350	CG5271	RpS27A
433	RE56673	CG2013	UbcD6
434	RE62259	CG31813	CG31813
435	RE59709	CG7939	RpL32
436	RE57333	CG11276	RpS4
437	RE60089	CG3167	MAN1
438	RH07443	CG8739	cmp44E
439	RH21608	CG1163	RpII18
440	RH29995	CG11271	RpS12
441	RH43519	CG7283	RpL10Ab
442	RH57501	CG2986	oho23B
443	RE29317	CG8025	Mtr3
444	RE01079	CG10305	RpS26
445	RE01365	CG8362	nmdyn-D7
446	RH12281	CG33110	CG33110
447	RH14840	CG8874	Fps85D
