# Supplementary material for: Ter94/VCP Is a Novel Component Involved in BMP Signaling
Source: PLoS One. 2014 Dec 3;9(12):e114475. doi: 10.1371/journal.pone.0114475 (PMC4255028; doi:10.1371/journal.pone.0114475)
Supplement: Table S2 — Candidate genes that are involved in BMP signaling after secondary screen. (PDF) [file pone.0114475.s002.pdf]

Table S2. Candidate genes that are involved in BMP signaling after secondary screen

Sample ID	DGC clone ID	Annotation ID (CG number)	Symbol
5	GH06563	CG7073	Sar1
6	GH11073	CG2267, CG10305	CG2267, RpS26
8	GH19261	CG14782	rush
10	GH13168	CG5778	CG5778
12	GH21935	CG5179	Cdk9
13	GH24939	CG17108	CG17108
16	GM27203	CG6163, CG11271	CG6163, RpS12
25	LP03545	CG7031	CG7031
28	LP06330	CG32638, CG11276	CG32638, RpS4
29	LP08442	CG3546	CG3546
30	LP11750	CG6041	CG6041
31	LP10071	CG1873, CG8280	EF1α100E, Ef1α48D
33	SD12691	CG11276	RpS4
35	SD17630	CG42788	CG42788
36	SD17528	CG9280	Glt
37	SD17959	CG3064	futsch
40	SD24044	CG5057	MED10
44	LD34409	CG5320	Gdh
46	LD32685	CG15817	CG15817
47	LP12034	CG2331	TER94
48	SD11109	CG17800	Dscam
51	LP23833	CG10514	CG10514
62	LD35644	CG1057	MED31
63	LD35705	CG7281	CycC
65	LD36256	CG17358	Taf12
72	SD10560	CG17183	MED30
78	LD46084	CG8609	Trap36
79	LP01553	CG5431	CG5431
81	LD22387	CG3180	RpII140
85	LD23157	CG5605	eRF1
89	LD21741	CG5537	CG5537
91	LD23744	CG7173	CG7173

93	GH01724	CG11139	p47
97	GH04632	CG32245	CG32245
98	GH09594	CG12582	CG12582
99	LD29131	CG7831	ncd
101	LD27504	CG15669	MESK2
102	LD27620	CG5466	CG5466
103	LD30122	CG5014	Vap-33-1
105	LD39166	CG4451	Hs6st
108	LD38749	CG30497	CG30497
109	LD39266	CG7073	sar1
111	LD44595	CG2621	sgg
115	GH12111	CG9696	dom
119	LD36178	CG6975	gig
122	LP01188	CG12104	CG12104
124	SD07852	CG4214	Syx5
127	GH14582	CG7776	E(Pc)
128	GH16721	CG6376	E2f
134	LD45157	CG4722	bib
136	LD45403	CG10084	CG10084
140	GH25238	CG14026	tkv
141	LD34489	CG30420	CG30420
143	SD10782	CG11661	Nc73EF
144	LD33331	CG7837	CG7837
145	GH23735	CG8205	fus
146	LD30271	CG15010	ago
150	GH01072	CG6884	Med21
153	LP01382	CG4204, CG7808	EloB, RpS8
155	LP04961	CG33113	Rtnl1
156	GM14481	CG11207	feo
157	LP12144	CG14206	RpS10b
162	LD46954	CG8370	CG8370
167	GH14384	CG9131	slmo
175	GH14380	CG12239	CG12239
177	GH09884	CG30384	CG30384
184	GH24286	CG9663	CG9663
186	LD44381	CG15532	hdc

187	SD02490	CG12031	MED14
189	LD25641	CG7282	CG7282
190	SD03094	CG9311	CG9311
191	LD46954	CG8370	CG8370
193	SD04853	CG17233	CG17233
194	GH01409	CG5166	Atx2
197	LD31537	CG9537	DLP
199	LD33277	CG15319	nej
200	LD31893	CG7904	put
204	LD30182	CG18734	Fur2
205	AT09438	CG4878	eIF3-S9
206	AT07769	CG7542	CG7542
208	AT13263	CG7003	CG7003
209	AT13773	CG5207	scpr-A
213	AT02695	CG2171	Tpi
215	AT05318	CG32319	CG32319
216	AT22129	CG15532	hdc
221	AT27578	CG9412	rin
223	AT23070	CG15515	CG15515
224	AT03646	CG6292	CycT
225	AT07244	CG2331	TER94
229	AT24649	CG14472	poe
233	GM02885	CG2331	TER94
234	LD06574	CG5183	KdelR
239	LD21089	CG8367	cg
240	GM05133	CG32555	RhoGAPp190
243	GM09915	CG15611	CG15611
244	GM01240	CG13900	CG13900
247	LD08906	CG4936	CG4936
249	LD19039	CG32000	CG32000
251	LD13191	CG17233	CG17233
255	LD07466	CG5634	dsd
257	LD09376	CG14792	sta
261	LD10287	CG12238	e(y)3
263	GM03914	CG7524	Src64B
264	GM04767	CG11180	CG11180

278	GM10051	CG10811	eIF-4G
279	GM10122	CG10215	Ercc1
280	LD01958	CG4330	CG4330
282	GM01970	CG4043	Rrp46
286	LD05303	CG32000	CG32000
289	LD03419	CG8416	Rho1
292	LD08529	CG7938	Sry-β
294	LD11064	CG1664	sbr
298	LD14913	CG8073	Pmm45A
303	RE01736	CG7890	Hs3st-B
305	RE01528	CG4563	CG4563
306	RE03692	CG5582	CG5582
311	RE05287	CG11125	CG11125
314	RE04130	CG15739	CG15739
317	RE10012	CG4158	wor
318	RE11206	CG18657	NetA
321	RE09982	CG9503	CG9503
348	RE39081	CG8254	exex
350	RE41410	CG7264	CG7264
352	RE43020	CG12017	CG12017
357	RE45331	CG43369	Mitf
361	RE54483	CG2033	RpS15Aa
363	RE55472	CG12265	CG12265
367	RE66843	CG3320	Rab1
371	RE68984	CG1873	Ef1alpha100E
372	RE69372	CG5333	trus
373	RE70632	CG1810	mRNA-cap
378	RE08425	CG7283	RpL10Ab
379	RE17389	CG7239	CG7239
383	RE72705	CG12399	Mad
384	RE75106	CG11357	CG11357
385	RH04426	CG4264	Hsc70-4
388	RH06886	CG7808	RpS8
391	RH07244	CG7808	RpS8
395	RH09938	CG10652	RpL30
396	RH12258	CG6619	CG6619

399	RH25914	CG1524	RpS14a
402	RH35331	CG1810	mRNA-cap
405	RH34416	CG13779	Sem1
420	RH69713	CG16799	CG16799
422	RH69856	CG14880	CG14880
433	RE56673	CG2013	UbcD6
437	RE60089	CG3167	MAN1
442	RH57501	CG2986	oho23B
